# Attenuated p53 activation in tumour-associated stromal cells accompanies decreased sensitivity to etoposide and vincristine

**DOI:** 10.1038/sj.bjc.6604465

**Published:** 2008-07-01

**Authors:** A C Dudley, S-C Shih, A R Cliffe, K Hida, M Klagsbrun

**Affiliations:** 1 Vascular Biology Program, Department of Surgery, Children's Hospital and Harvard Medical School, Boston, MA, USA; 2 Department of Pathology, Beth Israel Deaconess Medical Center and Harvard Medical School, Boston, MA, USA; 3 Department of Microbiology and Molecular Genetics, Harvard Medical School, Boston, MA, USA; 4 Hokkaido University, Grad. School of Dental Medicine and Oral Pathology, Sapporo, Japan; 5 Department of Pathology, Children's Hospital and Harvard Medical School, Boston, MA, USA

**Keywords:** tumour, tumour microenvironment, tumour stroma, p53, drug resistance

## Abstract

Alterations in the tumour suppressor p53 have been reported in tumour-associated stromal cells; however, the consequence of these alterations has not been elucidated. We investigated p53 status and responses to p53-activating drugs using tumour-associated stromal cells from A375 melanoma and PC3 prostate carcinoma xenografts, and a spontaneous prostate tumour model (TRAMP). p53 accumulation after treatment with different p53-activating drugs was diminished in tumour-associated stromal cells compared to normal stromal cells. Tumour-associated stromal cells were also less sensitive to p53-activating drugs – this effect could be reproduced in normal stromal cells by p53 knockdown. Unlike normal stromal cells, tumour stromal cells failed to arrest in G_2_ after etoposide treatment, failed to upregulate p53-inducible genes, and failed to undergo apoptosis after treatment with vincristine. The lower levels of p53 in tumour stromal cells accompanied abnormal karyotypes and multiple centrosomes. Impaired p53 function in tumour stroma might be related to genomic instability and could enable stromal cell survival in the destabilising tumour microenvironment.

Both cancer and healing wounds are characterised by increased proliferation and infiltration of endothelial cells, inflammatory cells, and fibroblasts (Dvorak, 1986). These infiltrating stromal cells (SC) may by tissue-resident and/or bone marrow-derived and together constitute the stromal microenvironment. The function of SC in tumours and physiological wound healing is to secrete matrix proteins and factors involved in tissue remodelling and to secrete chemotactic factors for inflammatory cells and endothelial cells. Thus, both tissue injury and cancer resemble an ‘activated’ state whereby the host's response is designed to heal the affected tissue (Coussens and Werb, 2002).

The tumour microenvironment consists of tumour cells, SC, and the matrix proteins, growth factors, and cytokines they produce (Liotta and Kohn, 2001). Fibroblasts comprise the majority of the tumour stroma, and their role in tumours is long-recognised (Seemayer *et al*, 1979). For example, fibroblasts are responsible for the synthesis of fibronectin and types I, III, and V collagens that make up basement membranes; they also secrete factors such as TGF-*β* that support nonautonomous tumour epithelial growth (Tlsty, 2001; Bhowmick *et al*, 2004). Functionally and phenotypically distinct myofibroblasts are also observed in most tumours (Sappino *et al*, 1988). Myofibroblasts express *α-*smooth muscle actin (*α*-SMA), which enhances their contractility and motility (Hinz *et al*, 2007), and they overexpress stromal-derived factor, which mobilises bone marrow-derived endothelial progenitor cells (Orimo *et al*, 2005).

Owing to genomic instability, cancer cells are typically mutable and develop drug resistance; thus, targeting SC in the tumour microenvironment may be a viable approach for helping to eliminate solid tumours (e.g., antiangiogenesis). However, it has recently become clear that like tumour cells, tumour-associated SC may be characterised by genomic instability and p53 mutations (Kurose *et al*, 2002; Hill *et al*, 2005). For example, numerous genetic amplifications and deletions were detected in murine stromal DNA isolated from implanted tumour xenografts (Pelham *et al*, 2006). Furthermore, cytogenetic (Moinfar *et al*, 2000; Allinen *et al*, 2004; Fukino *et al*, 2004; Hida *et al*, 2004) and epigenetic (Kaplan *et al*, 2005) alterations have been described in tumour stroma and in tissue adjacent to carcinoma (Deng *et al*, 1994). The possibility that cytogenetic alterations in tumour SC including p53 mutations might accompany changes in p53 function has not been addressed.

In cancer cells, the principal cause of resistance to chemotherapeutic drugs is chromosomal instability accompanied by deletion of or mutations in p53; however, both drug resistance and increased sensitivity in a p53 null background have been noted (Bunz *et al*, 1999; Burdelya *et al*, 2006). p53 is mutated or lost in about half of all cancers probably as a consequence of selection pressure for p53 mutations, which enable tumour cell survival (Gorgoulis *et al*, 2005). The importance of functional p53 in tumours has been formally proven by reintroduction of wild-type p53 in tumour cells *in vivo* (Martins *et al*, 2006; Ventura *et al*, 2007). However, it has been noted that re-imposition of p53 function was quickly mitigated by p53 inactivation and the emergence of p53-resistant tumours (Martins *et al*, 2006). Despite these detailed studies investigating p53 function in tumour cells, no studies to date have examined the relationship between p53 status and sensitivity to cytotoxic DNA-damaging agents in tumour-associated SC.

In this report, we show that tumour SC, in contrast to normal SC, fail to undergo growth arrest and apoptosis after treatment with etoposide or vincristine, respectively. Tumour SC show diminished p53 expression, and p53 fails to or only marginally accumulates after treatment with p53-activating stimuli. Tumour SC also have multiple centrosomes and are aneuploid – both of which are readouts of abnormal p53 function. Together, these results indicate that, similar to tumour cells, alterations in p53 function and decreased sensitivity to commonly used p53-activating chemotherapeutic drugs are features of tumour-associated SC.

## Materials and methods

### Cell lines and media

All primary cells were cultured in EGM2-MV medium (Cambrex Bioscience, Rockland, ME, USA) and were maintained in an atmosphere of 5% CO_2_ at 37°C. PC3MLN4 prostate carcinoma cells were grown in HAMS with 10% FBS, and A375SM melanoma cells were grown in MEM with 10% FBS. Tumour cells were grown under 10% CO_2_. Normal and tumour SC were obtained from isolated endothelial cultures overtaken by rapidly growing fibroblast-like cells. This was a common occurrence in our hands, and was unavoidable without strict monitoring of the pure endothelial cultures (Hida *et al*, 2004).

### Mice

All animal procedures were performed in compliance with Boston Children's Hospital Guidelines and were approved by the Institutional Animal Use and Care Committee. The procedures for cell isolation for A375 tumour xenografts were previously described (Hida *et al*, 2004). For the PC3MLN4 prostate carcinoma, one million cells were injected subcutaneously into the dorsal lateral flank of 8-week-old nu/nu mice. When tumours reached 1 cm^3^ (approximately 4–6 weeks postimplantation), tumours were harvested as described by Hida *et al* (2004). Tumours from five mice were combined for the cell isolation. TRAMP mice were genotyped at 4 weeks of age (Transnetyx, Cordova, TN, USA) and tumours were harvested when mice reached 20–22 weeks.

### Antibodies and reagents

The mouse monoclonal p53 and rabbit polyclonal pSER15 and pSER20 p53 antibodies were purchased from Cell Signaling Technologies (Danvers, MA, USA). *β*-actin, caldesmon, *α*-SMA, and fibroblast-specific protein-1 (FSP-1) antibodies were from Sigma-Aldrich (St Louis, MO, USA). The CD105 antibody was from BD Pharmingen (San Jose, CA, USA). Rabbit polyclonal pericentrin antibody was from Covance (Berkeley, CA, USA). Etoposide and vincristine were dissolved in DMSO and were purchased from Sigma-Aldrich.

### Drug treatment

To analyse p53 expression, cells were seeded at a density of 200 000 cells per 10 cm^2^ and left overnight. The next day, the indicated drug was added, and the cells were incubated between 8 and 24 h. Cell lysates were prepared in RIPA buffer and subjected to western blotting according to standard methods. Nuclear fractions were prepared using the NE-PER kit according to the manufacturer (Pierce, Rockford, IL, USA). For viability studies, cells were seeded at 2500 cells per well in a 96-well plate. The next day, the indicated concentration of each drug was added, and the cells were incubated for an additional 72 h. Cell counts were determined by dispersing the cells in trypsin and counted using a Coulter counter (Beckman Coulter, Fullerton, CA, USA; Model Z1).

### Fluorescence-activated cell sorting

Fluorescence-activated cell sorting (FACS) for cell characterisation was carried out on live cells used between 6 and 10 passages. Propidium iodide and annexin V staining were done according to the manufacturer's instructions (BD Pharmingen). Cells were analysed on a BD FACSCalibur System.

### Immunofluorescence

Cells were seeded at a density of 10 000 cells per well in gelatin-coated eight-well chamber slides. Confluent cells were washed twice with PBS and then fixed with ice-cold 100% methanol at −20°C for 20 min. The fixed cells were rinsed briefly with PBS and then blocked for 1 hour at room temperature with PBS containing 5% BSA. After blocking, antibodies were added overnight at 4°C in a humidified chamber. The next day, cells were rinsed with PBS and then blocked again for 30 min at room temperature. Secondary antibodies were added and the cells were incubated an additional hour at room temperature protected from light. Finally, the cells were washed with PBS and then mounted using Gel Mount (Biomeda, Foster City, CA, USA) containing 0.4 *μ*g ml^−1^ 4′,6-diamidino-2-phenylindole.

### p53 sequencing

cDNA was prepared from normal and tumour SC by reverse transcription. Primers specific for the mouse p53-coding regions were used to amplify a 1173 bp fragment that was subcloned into the PCR-2 vector (Invitrogen, Carlsbad, CA, USA). Plasmid DNA was cloned in Top10 cells, purified, and sequenced using the same primers for PCR amplification. Sequences were analysed using Chromas software.

### Karyotyping

Karyotyping was carried out by the Brigham and Women's Cytogenetics Core Facility, Boston, MA, USA.

### siRNA

Mouse siRNA for p53 was purchased from Dharmacon (Lafayette, CO, USA). Cells were transfected using Silentfect transfection reagent according to the manufacturer's instructions (BioRad, Hercules, CA, USA).

## Results

### Stromal cell characterisation

**Figure 1:**
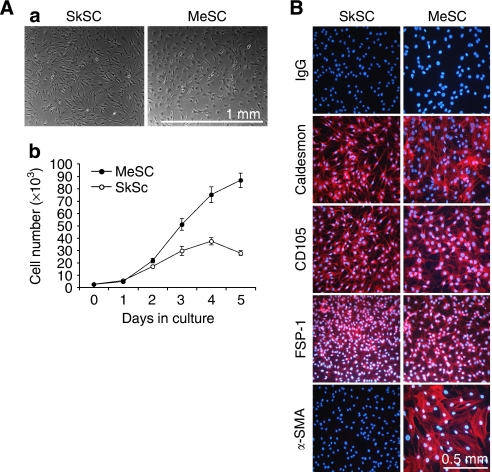
Stromal cell characterisation. (**A**) Morphology of SC from normal mouse skin or subcutaneous xenografts of A375 melanoma (**a**) and comparison of their growth properties in culture (**b**). (**B**) By immunofluorescence, SC stained uniformly positive for caldesmon, CD105, and FSP-1. *α*-SMA was expressed only in the tumour SC, indicative of a myofibroblast-like phenotype.

Previously, our laboratory identified cytogenetic alterations in tumour-specific endothelial cells (Hida *et al*, 2004). In the present study, we found that isolates of endothelial cells from A375 melanoma were frequently overtaken by fibroblast-like stromal cells (MeSC), which grew on average two to three times the rate of normal SC from mouse skin (SkSC) (Figure 1A). In contrast to tumour endothelial cells described by Hida *et al* (2004), these cells were negative for the EC markers CD31 and VEGFR2 (data not shown), but positive for caldesmon, CD105, and FSP-1 (Figure 1B). *α-*Smooth muscle actin expression was restricted to the SC from melanoma, possibly owing to their ‘activated’ or myofibroblast-like phenotype observed in most tumours (Orimo *et al*, 2005). On the basis of morphology and marker expression, the isolated SC used in this study were identified as fibroblasts or myofibroblasts.

### p53 function in normal and tumour stromal cells

**Figure 2:**
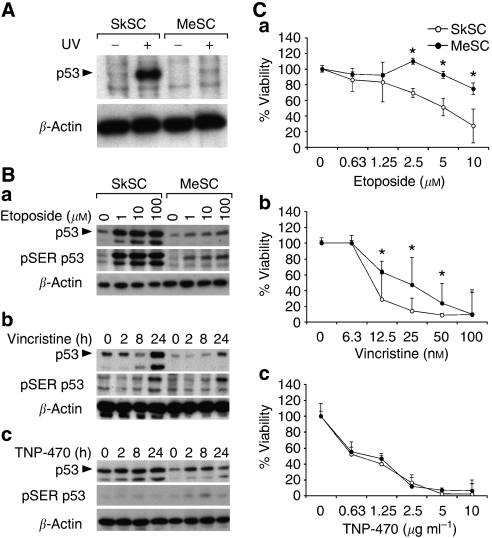
p53 function in tumour stromal cells. (**A**) Western blotting of whole-cell lysates from UV-treated SkSC and MeSC. Cells were treated with 100 mJ cm^−2^ for 5 min and lysates were prepared 6 h later. (**B**) Western blotting of whole-cell lysates from 8 h etoposide-treated (**a**), 1 nM vincristine-treated (**b**), or 10 *μ*g ml^−1^ TNP-470-treated cells (**c**). The same blots were stripped and re-probed with rabbit polyclonal pSER15 p53 antibodies and then mouse monoclonal *β*-actin antibodies. (**C**) Dose–response curves for etoposide (**a**), vincristine (**b**), and TNP-470 (**c**). Cells were plated in triplicate and treated with each drug for 72 h before dispersing in trypsin and counting. ^*^Results are statistically significant (*P*<0.05) by student's *t*-test.

Ultraviolet (UV) light causes DNA damage, which is repaired via a p53-dependent mechanism (Nelson and Kastan, 1994). As expected, p53 levels were increased in normal SkSC after UV treatment (Figure 2A). Strikingly, p53 only marginally accumulated in MeSC after treatment with UV light. Next, the p53 responses in SkSC or MeSC following treatment with different p53-activating drugs were determined. Although etoposide caused an approximate five-fold increase in p53 and SER15-phosphorylated p53 in SkSC at all doses, only a two-fold increase in p53 was evident in MeSC (Figure 2B). Time course experiments using vincristine produced similar results, with a maximum seven- to eightfold increase in p53 levels in SkSC after drug treatment and only a one- to twofold increase in MeSC. TNP-470, a compound not known as a p53 inducer, did not cause a remarkable upregulation of p53 or p53 phosphorylation at any time point in both SC.

### MeSC are less sensitive to p53-activating drugs

**Figure 3:**
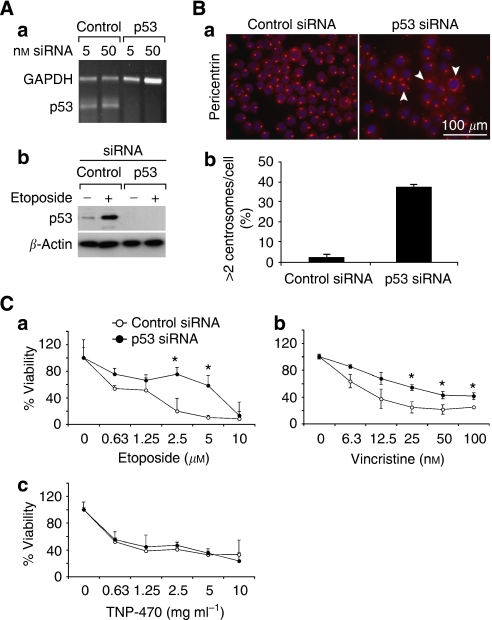
p53 knockdown in SkSC decreases sensitivity to etoposide and vincristine. (**A**) p53 knockdown was complete at the RNA (**a**) and protein levels (**b**) using siRNA. (**B**) p53 knockdown resulted in multiple less-uniform centrosomes (**a**), and about 30% of the cells had an abnormal >2 pericentrin signals per cell (**b**). (**C**) Cell numbers determined in p53 knockdown SkSC after a 72 h treatment with etoposide (**a**), vincristine (**b**), and TNP-470 (**c**). Cells were plated in triplicate and treated with each drug for 72 h before dispersing in trypsin and counting. ^*^Results are statistically significant (*P*<0.05) by student's *t*-test.

We compared the viability of SkSC and MeSC following treatment with each p53-activating drug. Although both cell types were growth-inhibited by etoposide and vincristine, MeSC were consistently less sensitive when compared to SkSC. In contrast, no difference in viability was seen in SkSC or MeSC treated with TNP-470, which also did not upregulate p53 (Figure 2C). To confirm a p53-specific effect, we knocked down p53 in SkSC using p53 siRNA. p53 knockdown was complete at the RNA and protein levels (Figure 3A). As a readout of p53 function, we measured centrosome numbers in SkSC after p53 knockdown (Bennett *et al*, 2004). p53 knockdown in SkSC resulted in multiple centrosomes per cell, consistent with the role of p53 in regulating centrosome duplication (Figure 3B). Furthermore, the dose–response curves for etoposide and vincristine showed that p53 knockdown rendered SkSC less sensitive to each drug (Figure 3C). No change in sensitivity to TNP-470 was observed, consistent with the failure of TNP-470 to activate p53.

### p53 localises to the nucleus in MeSC but the total cellular p53 pool is reduced

**Figure 4:**
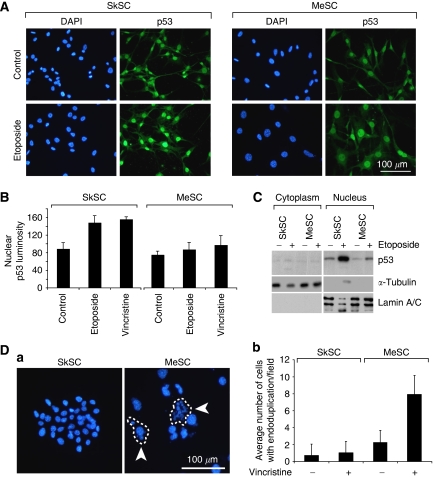
p53 localises to the nucleus in SkSC and MeSC, but the total cellular p53 pool is reduced. (**A**) p53 immunofluorescence in SkSC and MeSC treated with etoposide (10 *μ*M, 8 h). Note the predominant nuclear staining for p53 in both the cell types, although the p53 signal in MeSC is diminished. (**B**) The luminosity for the nuclear p53 signal in treated and untreated cells was measured in 10 cells from three random fields and plotted. (**C**) Western blots of purified nuclear and cytoplasmic fractions from etoposide-treated cells (10 *μ*M, 8 h). As loading controls, blots were stripped and re-probed with *γ*-tubulin (cytoplasm) and lamin A/C (nucleus). (**D**) Vincristine-treated cells (1 nM, 24 h) were methanol-fixed, and the nuclei stained with DAPI. The circled cells are single cells, and the arrows point to endoduplicated nuclei (**a**). Cells in 10 random fields were counted, and the average number of cells with multiple nuclei (>3 per cell) was plotted (**b**).

As p53 mutations may result in its sequestration in cytoplasm or nucleus, we determined the subcellular localisation of p53 by immunofluorescence and cell fractionation studies. By immunofluorescence, p53 localised predominately to the nucleus in both SkSC and MeSC in untreated cells and in cells treated with etoposide (Figure 4A). However, when the results were quantified, nuclear p53 levels in SkSC treated with etoposide or vincristine were approximately twofold higher compared to MeSC (Figure 4B). Western blotting of purified nuclear and cytosolic extracts of etoposide-treated cells confirmed that p53 was predominately localised to the nucleus, where it was strikingly increased in SkSC, but not MeSC (Figure 4C). It is worth noting that almost 10 times as many cells with endoduplicated nuclei (nuclear division without cell division) were detected in MeSC compared to SkSC after vincristine treatment (Figure 4D). Endoduplication is commonly observed in p53-null mouse embryonic fibroblasts treated with microtubule inhibitors and occurs due to checkpoint failure in the absence of normal p53 function (Lanni and Jacks, 1998).

### MeSC fail to arrest in G_2_ after etoposide treatment

**Figure 5:**
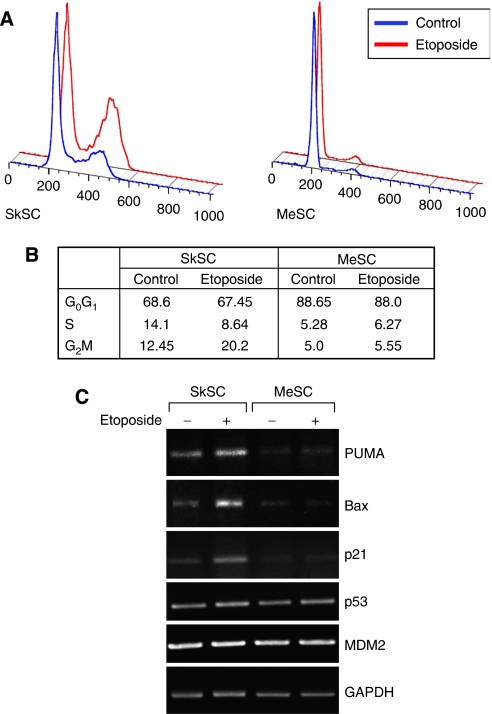
MeSC fail to arrest in G_2_ after etoposide treatment. (**A**) Cells treated with 1 *μ*M etoposide were analysed by FACS 24 h later. Note the larger G_2_ peak on the histogram in etoposide-treated SkSC but not in MeSC. (**B**) DNA histograms were analysed and the results from two experiments were averaged and are shown in the figure (numbers are per cent). (**C**) Semiquantitative RT–PCR analysis for p53-indicible genes in SC treated with 1 *μ*M etoposide for 24 h.

As the growth inhibitory effects of etoposide are mainly due to G_2_ arrest, cell cycle status in etoposide-treated cells was determined by propidium iodide (PI) staining followed by FACS. In SkSC treated with etoposide, the number of cells in G_2_ almost doubled compared to untreated cells. In contrast, MeSC failed to accumulate in G_2_ (Figure 5A and B). By semiquantitative RT–PCR, the expression of p53-inducible genes related to cell cycle arrest and apoptosis including PUMA, Bax, and p21 was upregulated in SkSC, but not in MeSC after etoposide treatment (Figure 5C). Notably, the constitutive levels of these genes were also qualitatively lower in MeSC relative to SkSC. No differences in p53 or in the negative regulator of p53 stability, MDM2, were detected. Taken together, these results were consistent with a failed induction of p53 and p53-inducible genes related to apoptosis or cell cycle arrest in MeSC after etoposide treatment.

### MeSC fail to undergo apoptosis after vincristine treatment

**Figure 6:**
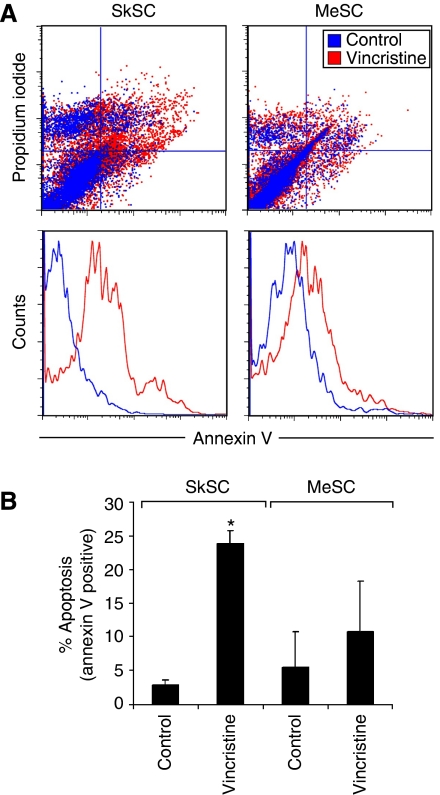
MeSC fail to undergo apoptosis after vincristine treatment. (**A**) Cells treated with 1 nM vincristine for 24 h were double stained with PI and AV and analysed by FACS. An increase in PI^+^/AV^+^ and PI^−^/AV^+^ cells was detected in vincristine-treated SkSC compared to MeSC. (**B**) The average numbers of early apoptotic AV^+^ cells from two experiments were plotted. ^*^Results are statistically significant (*P*<0.05) by student's *t*-test.

Dual staining using PI and annexin V (AV) in vincristine-treated cells indicated an increase in AV^+^/PI^+^ cells in SkSC, but not MeSC (Figure 6A). The number of early apoptotic AV^+^ cells increased six times above untreated cells in SkSC but only two times in MeSC (Figure 6B). Thus, the decreased sensitivity to vincristine in MeSC relative to SkSC was most likely due to decreased p53-dependent apoptosis.

### p53 function is impaired in stromal cells from PC3 and TRAMP prostate tumours

**Figure 7:**
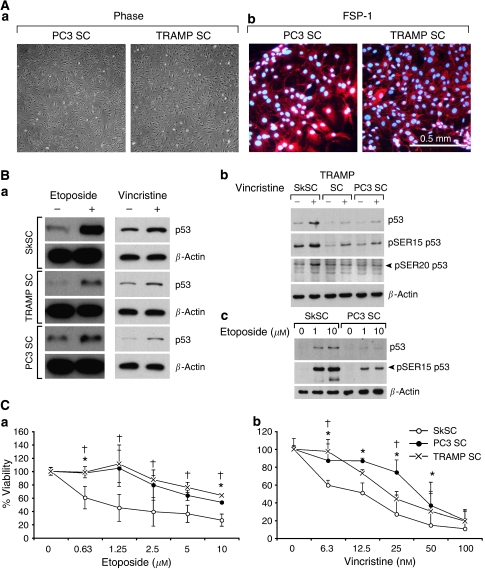
p53 function is impaired in stromal cells from PC3 and TRAMP prostate tumours. (**A**) Both PC3SC and TRAMPSC had a fibroblast-like morphology (**a**) and uniformly expressed FSP-1 (**b**). (**B**) After treatment with etoposide (10 *μ*M, 8 h) or vincristine (1 nM, 24 h), p53 accumulation was diminished in TRAMPSC and PC3SC compared to normal SkSC (**a**). Western blotting for pSER15 and pSER20 after vincristine (**b**) (1 nM, 24 h) or etoposide (8 h) treatment (**c**). Blots were striped and re-probed with p53 or *β*-actin antibodies. (**C**) Viabilities of TRAMPSC and PC3SC after treatment with etoposide (**a**) or vincristine (**b**). Cells were plated in triplicate and treated with each drug for 72 h before dispersing in trypsin and counting. Asterisk (^*^) indicates results are statistically significant (*P*<0.05) by Student's *t*-test when comparing SkSC *vs* PC3 SC and a dagger (†) when comparing SkSc *vs* TRAMP SC.

To determine if SC from different tumours also showed diminished p53 function, we isolated SC from PC3 xenografts and spontaneous prostate tumours in TRAMP mice. Both PC3SC and TRAMPSC had a fibroblast-like morphology, were negative for EC markers (data not shown), and were positive for FSP-1, indicating that they were tumour-associated fibroblasts, similar to SC from A375 melanoma (Figure 7A). For all tumour xenografts used in this study, diphtheria toxin was used to eliminate human tumour cells from cultures (Arbiser *et al*, 1999). Diphtheria toxin could not be used in the TRAMP model because these are spontaneous tumours with no human component. However, TRAMPSC did not express epithelial-specific E-cadherin by FACS or immunofluorescence, indicating absence of contaminating tumour cells (data not shown). Similar to MeSC, both PC3SC and TRAMPSC showed only modest increases in p53 after treatment with etoposide or vincristine, whereas p53 levels in SkSC were markedly upregulated (Figure 7B). Both vincristine and etoposide treatment increased SER15 phosphorylation in both normal and tumour SC, although the levels were reduced in tumour SC presumably due to the decrease in total p53 levels. However, although SER20 was phosphorylated in normal SC after vincristine treatment, no increase in SER20 phosphorylation was evident in tumour SC. No SER20 phosphorylation was detected in SkSC or PC3SC after treatment with etoposide (data not shown). Similar to MeSC, and in good accord with the diminished p53 levels, TRAMPSC and PC3SC were less sensitive to etoposide and vincristine compared to SkSC (Figure 7C). These results suggest that tumour-associated SC from different tumour types show diminished p53 protein levels and impaired p53 function.

### Tumour stromal cells have multiple centrosomes and aneuploid karyotypes

**Figure 8:**
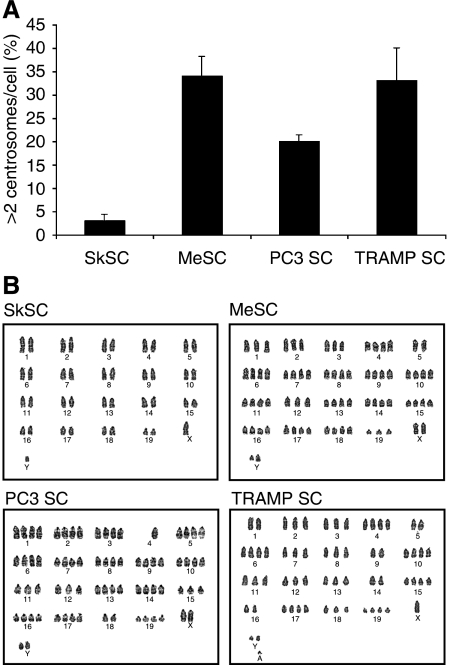
Tumour stromal cells have multiple centrosomes and aneuploid karyotypes. (**A**) Centrosomes were labeled with pericentrin antibodies and counted. One hundred cells were scored and averaged from three different fields. (**B**) Karyotypes for SkSC, MeSC, PC3SC, and TRAMPSC showing aneuploidy in all tumour SC, whereas SkSC were normal.

Hida *et al* (2004) recently reported abnormal centrosomes and abnormal karyotypes in tumour-specific endothelial cells. About 30% of MeSC, TRAMPSC, and PC3SC also had abnormal multiple centrosomes by pericentrin staining (Figure 8A). Karyotypes on the isolated cells showed that all tumour SC had heterogeneous aneuploid chromosomes whereas SkSC were normal (Figure 8B). Taken together, SC from different types of tumours (melanoma *vs* prostate) and different models (xenograft *vs* spontaneous) are characterised by aneuploid karyotypes and multiple centrosomes.

## Discussion

Our study provides evidence for impaired p53 function in tumour-associated SC. All tumour SC examined were characterised by diminished p53 protein levels, decreased sensitivity to cytotoxic drugs, and genomic instability indicated by multiple centrosomes and aneuploid karyotypes. Although the nature of the p53 defect in tumour SC is not yet clear, impaired p53 function in tumour stroma could enable SC survival in the tumour microenvironment and contribute to genomic instability.

Host SC can constitute a significant percentage of the total tumour bulk as shown in GFP-SCID mice implanted with tumour xenografts (Udagawa *et al*, 2006). Tumour-associated fibroblasts or mesenchymal-like cells comprise a large portion of the tumour stroma. Collectively, these cells might arise from tissue-resident activated fibroblasts, tumour epithelial cells undergoing epithelial-to-mesenchymal transition, resident stem or mesenchymal-like cells, or bone marrow-derived progenitors. Recently, mesenchymal stem cells localised to breast carcinoma were shown to increase metastatic potency (Karnoub *et al*, 2007). Thus, by providing scaffolds for tumour cells and other SC, and by producing growth factors and chemotactic factors for inflammatory cells and vascular progenitors, tumour SC can enable tumour growth and possibly metastasis (Olumi *et al*, 1999; Bhowmick *et al*, 2004). Targeting tumour SC may therefore be a viable approach for eliminating solid tumours (Hofmeister *et al*, 2008).

Recently, our laboratory reported that endothelial cells from human-to-mouse xenografts were aneuploid with centrosome abnormalities 4–6 weeks after implantation (Hida *et al*, 2004). Additional studies have confirmed both cytogenetic and epigenetic alterations in tumour-associated SC and tissue adjacent to carcinoma (Deng *et al*, 1994; Moinfar *et al*, 2000; Streubel *et al*, 2004). The mechanism(s) of these cytogenetic changes in tumour stroma are not yet clear. One possibility is that SC and tumour cells may fuse, as hypothesised in cases of allelic imbalance and genomic instability in human breast stroma (Weber *et al*, 2006). However, we found no evidence of human DNA in the karyotypes of murine tumour SC from human-to-mouse xenografts. Selection pressure for SC with diminished p53 function, and thus a survival advantage in the tumour microenvironment, is also a possibility. For example, nonautonomous oncogenic stress in tumour epithelium may result in the selection of SC with LOH at the p53 locus (Lu *et al*, 2001; Hill *et al*, 2005; Kiaris *et al*, 2005). It is likely that impaired p53 function in tumour SC might enable the propagation of cells with damaged DNA due to checkpoint failure leading to chromosomal instability.

A potential caveat related to targeting tumour SC is the loss of function of tumour suppressor genes in the stromal compartment (Patocs *et al*, 2007). Hill *et al* (2005) described widespread p53 loss in tumour mesenchyme after 20–25 weeks in a prostate cancer model. We evaluated p53 function and performed karyotypes in TRAMPSC at 22 weeks following tumour initiation and in SC from tumour xenografts at 4–6 weeks postimplantation. In either case, alterations in p53 function, diminished p53 protein levels and abnormal karyotypes were evident, irrespective of time or tumour model. At present, we have been unable to detect mutations in the p53-coding regions in SC, nor could we detect p53 LOH by real-time–PCR using genomic DNA (data not shown). Although real-time–PCR is a sensitive and well-established method to detect LOH, heterogeneity in p53 status in the cultured tumour SC could confound these results. Therefore, LOH may only be evident on a cell-to-cell basis. Further studies will be needed to address the mechanism of impaired p53 function in tumour SC in our *in vitro* system and whether tumour SC can acquire drug resistance *in vivo*.

It is also possible that alterations in p53-interacting proteins including the kinases responsible for p53 phosphorylation could contribute to impaired p53 function in tumour SC. For example, DNA damage imparts a well-characterised phosphorylation of SER15 and SER20 in the p53 transcriptional activation domain. Phosphorylation of these residues is thought to inhibit the interaction of p53 with MDM2 and increase its stability (Appella and Anderson, 2001). Although lower levels of p53 SER15 phosphorylation were evident in tumour SC in this study, this was most likely due to the decrease in total levels of the p53 protein. On the other hand, SER20 did not appear to be phosphorylated in tumour SC relative to normal SC after vincristine treatment. It remains possible that in tumour SC, alterations in pathways secondary to the stability of p53, rather than direct alterations in p53, could impart a destabilising effect on p53 leading to its degradation.

The idea that tumour SC can contribute to tumour growth and perhaps metastasis is an emerging concept in cancer biology (Karnoub *et al*, 2007). Though alterations in p53 in tumour SC have been shown previously *in vivo*, we show that diminished p53 function accompanies genomic instability and decreased sensitivity to cytotoxic drugs.
